# Centrosome amplification is a frequent event in circulating tumor cells from subjects with metastatic breast cancer

**DOI:** 10.1002/1878-0261.12687

**Published:** 2020-05-19

**Authors:** Ashok Singh, Ryan A. Denu, Serena K. Wolfe, Jamie M. Sperger, Jennifer Schehr, Tessa Witkowsky, Karla Esbona, Richard J. Chappell, Beth A. Weaver, Mark E. Burkard, Joshua M. Lang

**Affiliations:** ^1^ Carbone Cancer Center University of Wisconsin‐Madison WI USA; ^2^ Department of Medicine Division of Hematology/Oncology University of Wisconsin‐Madison WI USA; ^3^ Departments of Statistics and of Biostatistics & Medical Informatics University of Wisconsin‐Madison WI USA; ^4^ Department of Cell and Regenerative Biology and Department of Oncology McArdle Laboratory for Cancer Research University of Wisconsin‐Madison WI USA

**Keywords:** breast cancer, centrin, centrosome amplification, CTC, EpCAM, pericentrin

## Abstract

Centrosome amplification (CA) is a common phenomenon in cancer, promotes genomic stability and cancer evolution, and has been reported to promote metastasis. CA promotes a stochastic gain/loss of chromosomes during cell division, known as chromosomal instability (CIN). However, it is unclear whether CA is present in circulating tumor cells (CTCs), the seeds for metastasis. Here, we surveyed CA in CTCs from human subjects with metastatic breast cancer. CTCs were captured by CD45 exclusion and selection of EpCAM‐positive cells using an exclusion‐based sample preparation technology platform known as VERSA (versatile exclusion‐based rare sample analysis). Centriole amplification (centrin foci> 4) is the definitive assay for CA. However, determination of centrin foci is technically challenging and incompatible with automated analysis. To test if the more technically accessible centrosome marker pericentrin could serve as a surrogate for centriole amplification in CTCs, cells were stained with pericentrin and centrin antibodies to evaluate CA. This assay was first validated using breast cancer cell lines and a nontransformed epithelial cell line model of inducible CA, then translated to CTCs. Pericentrin area and pericentrin area x intensity correlate well with centrin foci, validating pericentrin as a surrogate marker of CA. CA is found in CTCs from 75% of subjects, with variability in the percentage and extent of CA in individual circulating cells in a given subject, similar to the variability previously seen in primary tumors and cell lines. In summary, we created, validated, and implemented a novel method to assess CA in CTCs from subjects with metastatic breast cancer. Such an assay will be useful for longitudinal monitoring of CA in cancer patients and in prospective clinical trials for assessing the impact of CA on response to therapy.

## Graphical abstract

Circulating tumor cells (CTCs) are a rare population of cancer cells released into peripheral circulation from primary and metastatic cancer sites. Centrosome amplification (CA) has been reported in virtually all human cancers and has been shown to make cells more invasive. In this study, we develop an assay to detect CA in CTCs. We find that CA is prevalent in CTCs from subjects with metastatic breast cancer.

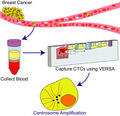

**Abbreviations**
CA: centrosome amplification
CTC: circulating tumor cell
PBMC: peripheral blood mononuclear cell
PLK4: polo‐like kinase 4
PMP: paramagnetic particles
VERSA: versatile exclusion‐based rare sample analysis

## 1 Introduction

Despite major advances in understanding the genetic, molecular, and cellular underpinnings of human cancer, metastatic disease remains incurable and accounts for the majority of cancer deaths. Genomic instability is a hallmark of human cancers (Gordon *et al.*, 2012) and, when it occurs at the level of whole chromosomes, yields an alteration of cellular chromosomal content. One‐time errors in chromosome segregation during cell division result in aneuploidy, an abnormal chromosomal content in daughter cells. When errors in chromosome segregation persist in multiple divisions, aneuploidy varies between cells and over time, a phenomenon known as chromosomal instability (CIN). CIN is a common feature of cancer, which led to the hypothesis that it can promote tumorigenesis. However, it has been found that aneuploidy can promote tumors, suppress tumors, or do neither, depending on the rate of CIN (Denu *et al.*, 2016; Funk *et al.*, 2016; Silk *et al.*, 2013; Weaver *et al.*, 2007; Zasadil *et al.*, 2016). Whereas low rates of CIN can weakly promote tumor growth, high rates cause cell death and suppress tumors (de Cárcer *et al.*, 2018; Funk *et al.*, 2016; Janssen *et al.*, 2009; Maia *et al.*, 2015; Rowald *et al.*, 2016). Preliminary evidence suggests that CIN may confer enhanced sensitivity to taxanes, widely used chemotherapy agents that interfere with microtubule dynamics (Janssen *et al.*, 2009; Maia *et al.*, 2015; Zasadil *et al.*, 2014). Although the mechanistic underpinnings of CIN are incompletely known, centrosome amplification (CA) is a common and readily detectable cause of CIN (Denu *et al.*, 2016; Ganem *et al.*, 2009; Silkworth *et al.*, 2009).

The centrosome is the primary microtubule‐organizing center of the cell and helps control cellular polarity, migration, and division (Merdes and Cleveland, 1997). The potential mechanisms leading to CA include cell doubling (e.g., cytokinesis failure and cell–cell fusion) or over duplication of centrioles (Denu *et al.*, 2018; Godinho *et al.*, 2009; Holland *et al.*, 2010). CA occurs in multiple cancer types (Chan, 2011), including breast (Lingle *et al.*, 1998), where CA is associated with higher tumor stage, worse outcomes, and invasive features (Denu *et al.*, 2016). Further, CA increases cellular invasiveness (Godinho *et al.*, 2014), suggesting that tumors with CA could have greater metastatic potential; however, this has not been demonstrated in human subjects.

Circulating tumor cells (CTCs) are a rare population of tumor cells released into peripheral circulation from primary and metastatic tumor sites that may both contribute to the development of metastatic disease and reflect the heterogeneity that likely exists between various tumor deposits (Mundy, 2002). Further, the number of CTCs in peripheral blood has been shown to have prognostic information in both early and advanced disease, (Bidard *et al.*, 2014; Janni *et al.*, 2016) and these cells are thought to be at least in part responsible for metastasis and resistance to chemotherapy (de Bono *et al.*, 2008; Pukazhendhi and Glück, 2014). Using CTCs as a biomarker affords the advantage of capturing cells that are biologically relevant to the metastatic process.

To date, we can find no reports of CA in CTCs. In the present study, we developed and validated an automated method to assess CA in CTCs from patients with metastatic breast cancer. This method may serve as a minimally invasive tool to measure CA, which occurs in CTCs from a substantial fraction of subjects with metastatic breast cancer. Importantly, this assay for CA may serve as a predictive biomarker in the future for therapeutic response to agents whose efficacy is affected by CA or CIN.

## 2 Materials and Methods

### 2.1 Cell culture

CAL‐51 cells were obtained from DSMZ, and MCF7 and MDA‐MB‐231 cells were obtained from ATCC. Cell lines were validated by short tandem repeat (STR) analysis in 2015 with the University of Wisconsin Translational Initiatives in Pathology (TRIP) laboratory. Cell lines were grown in DMEM with 10% fetal bovine serum FBS and 1% pen/strep solution at 37^0^C and 5% CO_2_. Doxycycline‐inducible RPE1 cells were obtained from Dr. David Pellman (Harvard) and validated by assessing centrioles before/after addition of doxycycline (Godinho *et al.*, 2014). These cells were grown in DMEM:F12 media with 10% FBS and 1% pen/strep solution. To induce PLK4 overexpression and CA, RPE1 cells were treated with doxycycline (10 µg·mL^−1^) for 48 h. Mycoplasma testing was performed on all cell lines using R&D Systems Mycoprobe Mycoplasma Detection Kit with help from the University of Wisconsin Small Molecule Screening and Synthesis Facility.

### 2.2 Collection and processing of subject blood samples

The Institutional Review Board (IRB) at the University of Wisconsin–Madison approved this study (IRB #2014‐1214 and #H2009‐0019). After written informed consent was obtained, blood specimens were collected in vacutainer tubes (BD Biosciences) with EDTA anticoagulant and used for CTC enumeration and CA analysis. A total of 15 mL of blood was processed from each subject. Nucleated cells were isolated using Ficoll‐Paque gradient, and CD45‐negative fraction was isolated and loaded into a VERSA (versatile exclusion‐based rare sample analysis) microfluidic device, as detailed previously (Sperger *et al.*, 2016). Briefly, the CD45‐depleted cells were collected in input wells and incubated with biotinylated EpCAM linked paramagnetic particles (PMPs) from the Dynabeads FlowComp Flexi kit (Life Technologies) for 30 min at 4°C in a rotating tumbler. After 30 min, EpCAM‐captured epithelial cells were transferred to a staining well and incubated with Alexa Fluor 647‐conjugated antibodies (CD45, CD34, CD11b, and CD14) and Hoechst dye for 30 min at 4°C. Cells were released from PMPs using FlowComp release buffer performed for 5–10 min at room temperature. Laboratory investigators were blinded to the clinical–pathological status of the subjects during CTC evaluation.

### 2.3 MCF7 cell capture and spike‐in experiment

MCF7 cells were incubated for 10 min with 2 mm calcein AM (Life Technologies) in cell culture media. The cells were centrifuged and washed once in PBS, counted with a hemocytometer, and then re‐suspended in PBS. Cells were spiked in VERSA devices with PBS, 0.1 % BSA, and 2 mm EDTA. Percent of cells left behind in each well compared to the input number of cells was measured by placing approximately 1000 MCF7 cells in the input well. Cells were imaged in the input capture well before binding to EpCAM‐labeled PMPs in the VERSA device. Cells were counted manually.

To quantify assay sensitivity, we performed spike‐in experiments using MCF7 cells. MCF7 cells were serially diluted in PBS into approximately 5 cells, 100 cells, and 500 cells per 10 μL. To ensure accurate dilutions and to obtain a starting MCF7 cell number, 10 μL of each dilution was added to 4 glass isolator wells with PBS and Hoechst. After a 30‐min incubation, the isolator wells were imaged. Hoechst‐positive cells were counted and averaged to get a starting value for each of the 3 conditions. The 5‐cell dilution had a mean of 3.25 cells/10 μL, the 100‐cell dilution had 104.5 cells/10 μL, and the 500‐cell dilution had 658.5 cells/10 μL. MCF7s were spiked into VERSAs with PBMCs from healthy donors and processed in the VERSA according to the protocol detailed above. MCF7s were identified as being Hoechst+/Cytokeratin+/Exclusion‐. Capture efficiency percentages were determined by taking the number of MCF7s remaining after all of the wash, fixation, and permeabilization steps over the mean number of MCF7s in 10 μL of the original dilutions.

### 2.4 Automated quantification of pericentrin

EpCAM‐captured CTCs were fixed in methanol for 10 min at − 20 °C and stained with cytokeratin‐790 and primary antibodies against pericentrin overnight in sieve well using a humidified chamber at 4 °C. Cells were then washed with PBST (PBS + 0.1% Tween) and PBS‐BSA (0.1%) buffers and incubated with secondary antibody (donkey anti‐rabbit IgG 488) for 2 h at RT. Cells were washed with PBST and PBS‐BSA buffer solutions in the sieve wells of VERSA. Cells were transferred to glass coverslips with adhesive silicon isolator (electron microscopy sciences, Cat#70346‐44) and imaged (Hoechst, pericentrin, CK, exclusion channel, bright field).

Background subtraction was performed for each channel using rolling ball diameter of 10 Nikon NIS‐Elements version 4.51.01), and spot detection module was utilized to identify and detect the nuclei (Hoechst). Binary editor was used to review and edit the cell identification binary layer. A permanent binary layer was developed for CTC analysis. Cytokeratin^+^/Hoechst^+^/CD45^−^ cells were identified as CTCs. Pericentrin area and intensity were assessed by manually drawing a binary layer representing pericentrin foci on identified cells. The area and intensity were assessed for the encircled area of pericentrin for all CTCs and peripheral blood mononuclear cells (PBMCs) using NIS‐Elements.

The following antibodies were used: DSBX‐labeled anti‐EpCAM/TROP1 (R&D systems, AF960); pan‐cytokeratin (Abcam, Cambridge, UK, ab7753); Alexa Fluor 647‐conjugated anti‐CD11b (BioLegend, San Diego, CA, USA, 101218), Alexa Fluor 647‐conjugated anti‐CD14 (BioLegend, 325612); Alexa Fluor 647‐conjugated anti‐CD45 (BioLegend, 304008); Alexa Fluor 647‐conjugated anti‐CD34 (BioLegend, 343618); anti‐pericentrin (Abcam, ab4448, 1: 1000); and Alexa Fluor 488 donkey anti‐rabbit IgG (BioLegend, 406416).

### 2.5 Manual quantification of centrioles

For analysis of centrioles, the EpCAM‐captured cells were cytospun (1000g for 5 min) onto number 1.5, 13mm coverslips pretreated with poly‐L‐lysine (1mg·mL^−1^ poly‐L‐lysine, Sigma Aldrich, St. Louis, MO, USA) for 1 h, washed 5 times with deionized water, and allowed to dry. Cells were then fixed with 100% ice‐cold methanol for 15 min. Fixed cells were blocked for 30 min in 3% BSA and 0.1% Triton X‐100 in PBS (PBSTx + BSA). Primary antibodies were incubated in PBSTx + BSA for 1 h at RT and washed three times in PBSTx, followed by secondary antibody incubation in PBSTx + BSA for 30 min at RT and two washes with PBSTx. Cells were counterstained with DAPI and mounted on glass slides with Prolong Gold antifade medium (Invitrogen). Cells were stained for centrin (Millipore, 04‐1624), pan‐cytokeratin (Abcam, ab7753), and exclusion markers (see above). Pictures were taken using the nis‐elements ar microscope imaging Software (Nikon, Melville, NY, USA) in Nikon Eclipse Ti‐E with ORCA‐Flash 4.0 Digital CMOS camera (Hamamatsu) florescent microscope with 10‐20X objectives in all the channels. Centrin foci were manually counted under 100X magnification and scored in CTCs and PBMCs.

### 2.6 Statistical analysis

Data were analyzed using GraphPad Prism software (version 6). Standard t‐tests were performed to compare CA in CTCs versus PBMCs. Differences with P‐values ≤ 0.05 are considered significant.

## 3 Results

### 3.1 Characteristics of pericentrin reliably estimate centriole amplification in breast cancer cell lines

**Fig. 1:**
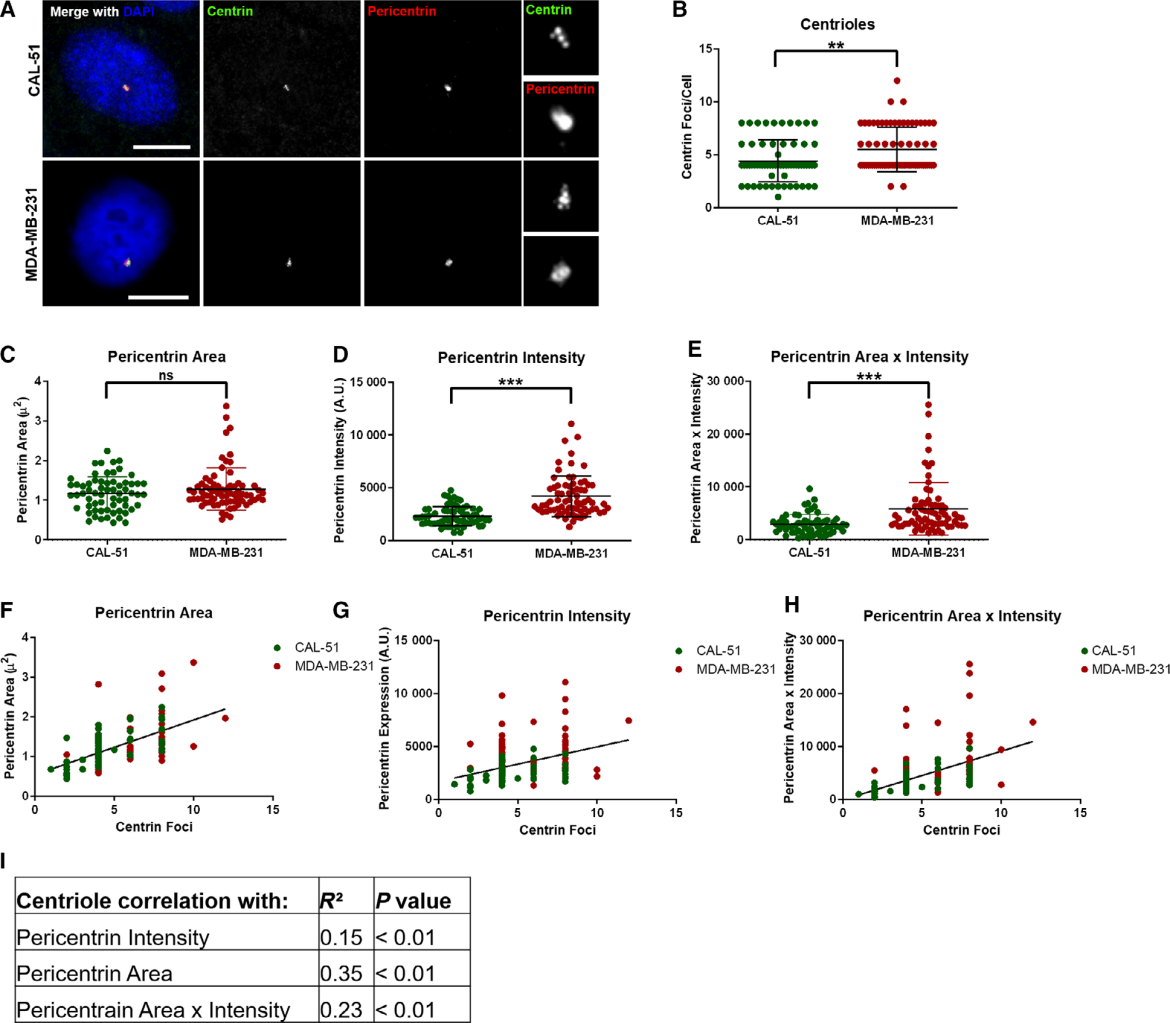
Centrosome size is the best predictor of centriole number in breast cancer cell lines. (A) Representative immunofluorescence images of CAL‐51 and MDA‐MB‐231 breast cancer cell lines. Scale bars = 10 μm. The smaller images on the right of the panel are enlargements of the centrosome. (B) Quantification of centrin foci. (C) Quantification of pericentrin area. (D) Quantification of pericentrin intensity. (E) A composite variable was created using the product of pericentrin area and intensity. (F–H) Correlation of centrin foci with pericentrin area (F), pericentrin intensity (G), or pericentrin area x intensity (H). (I) This table summarizes the correlation coefficients and statistical significance of these correlations. Throughout the figure, each dot represents one cell and bars represent mean ± SD.

The gold standard for assessing CA is by centriole enumeration using centrin staining (Denu *et al.*, 2018); however, this is technically challenging and cumbersome, particularly on patient samples, because it requires manual identification of CTCs and enumeration of centrioles. To develop a high‐throughput, objective, and repeatable method to assess CA, we correlated centrioles (manual enumeration of centrin foci) with more readily attainable centrosome parameters, namely pericentrin size and intensity, as these have been previously shown to correlate with CA (Lingle and Salisbury, 1999). The CAL‐51 and MDA‐MB‐231 cell lines were used as models of low and high CIN, respectively. We performed both manual counting of centrin foci (gold standard) and automated analysis of pericentrin features. As expected, MDA‐MB‐231 cells exhibited a significantly greater degree of CA than CAL‐51 cells (Fig. 1A,B). MDA‐MB‐231 cells also showed increases in pericentrin intensity, area, and area x intensity (Fig. 1C–E). All three measures of pericentrin were significantly correlated with centriole number (Fig. 1F–I), with pericentrin area showing the best correlation in this paired cell analysis. We conclude that high‐throughput assessment of pericentrin correlates well with centriole number.

### 3.2 Reliable detection of CA in VERSA‐captured cells

**Fig. 2:**
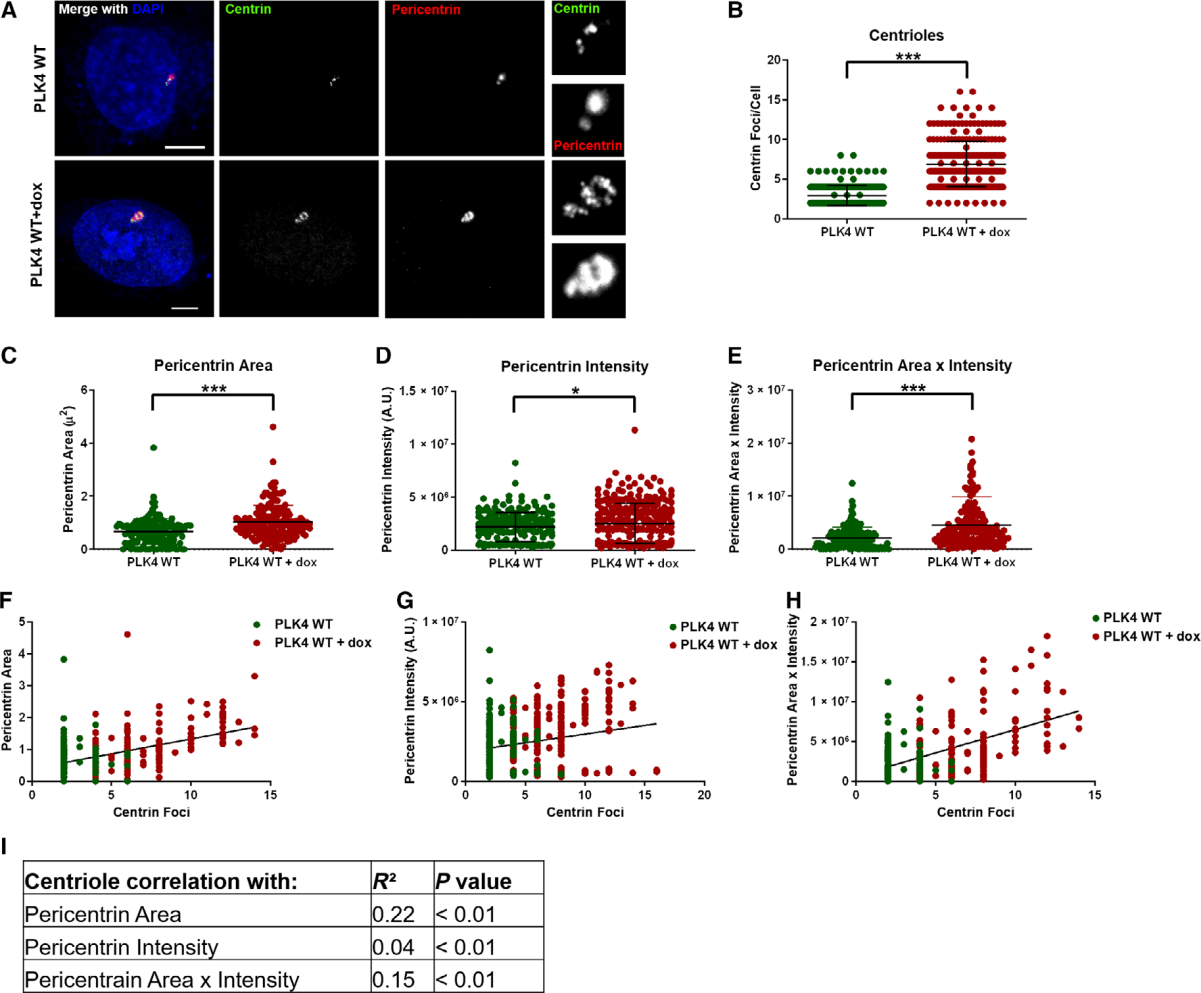
Centrosome size is the best predictor of centriole number in PLK4‐overexpression model of CA. (A) Representative immunofluorescence images of RPE1 cells with doxycycline‐inducible PLK4. The smaller images on the right of the panel are enlargements of the centrosome. Scale bars = 5 μm. (B) Quantification of centrin foci. (C) Quantification of pericentrin area. (D) Quantification of pericentrin intensity. (E) A composite variable was created using the product of pericentrin area and intensity. (F–H) Correlation of centrin foci with pericentrin area (F), pericentrin intensity (G), or pericentrin area x intensity (H). (I) This table summarizes the strength and statistical significance of these correlations. Throughout the figure, each dot represents one cell and bars represent mean ± SD.

To further validate our assay for assessing CA in CTCs, we used a doxycycline‐inducible PLK4 RPE1 cell line as an *in vitro* model system (Fig. 2A). In these cells, PLK4 overexpression induces CA (Godinho *et al.*, 2014). We induced PLK4 overexpression with doxycycline treatment and analyzed pericentrin intensity, pericentrin area, and centrin foci in fixed cells. In this RPE1 cell model, doxycycline‐inducible overexpression of PLK4 significantly increases centrin foci, pericentrin intensity, pericentrin area, and the composite variable (product of pericentrin area and intensity) (Fig. 2B–E). Similarly to breast cancer cell lines, centriole number correlates with all three measurements of pericentrin (Fig. 2F–I), with pericentrin area showing the best correlation.

### 3.3 VERSA reliably detects CA in inducible PLK4 RPE1 model

**Fig. 3:**
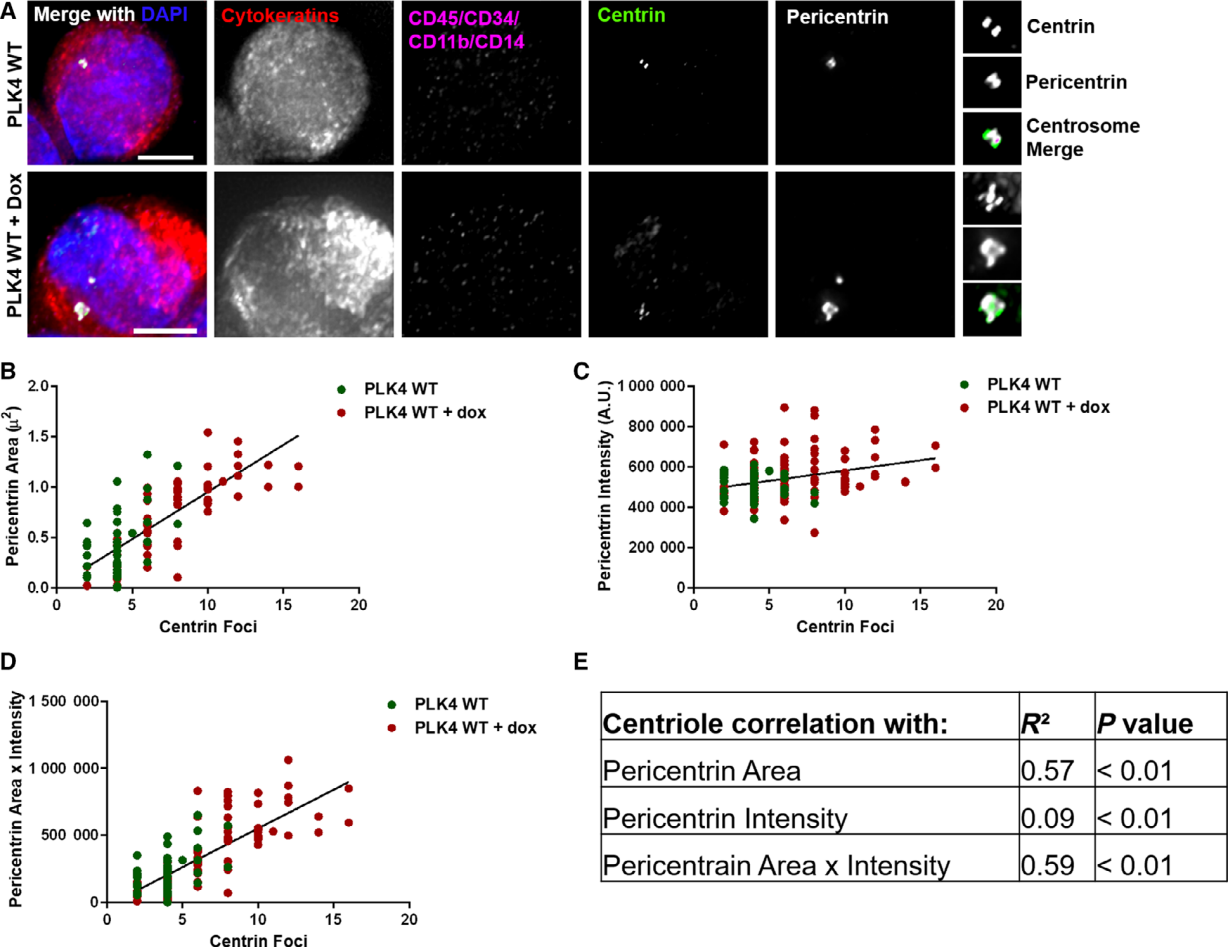
Assessment of CA using the VERSA platform. (A) Doxycycline‐inducible PLK4 RPE1 cells were run through the VERSA platform and analyzed. Representative images of pericentrin and centrin staining and imaging are shown. Scale bars = 10 μm. The smaller images on the right of the panel are enlargements of the centrosome. (B–D) Correlation of centrin foci with pericentrin area (B), intensity (C), and area x intensity (D). (E) This table summarizes the strength and statistical significance of these correlations. Throughout the figure, each dot represents one cell and bars represent mean ± SD.

We previously described the VERSA microfluidic platform, which reliably captures circulating tumor cells (CTCs) from blood in subjects with metastatic cancer (Sperger *et al.*, 2016). Using this method, blood is taken from subjects with metastatic cancer, processed by exclusion of CD45 + cells and capturing of EpCAM + cells in the VERSA, and stained for Hoechst, cytokeratin, and exclusion markers (CD11b, CD45, CD14, CD34). EpCAM^+^ cells were then stained with additional antibodies. In CTCs, pericentrin staining was done in the VERSA using half the cells, whereas the remaining half of cells were cytospun and stained on coverslips to assess centrin. CTCs were defined as positive for cytokeratin and negative for the exclusion markers. To verify that CA could be reliably detected using this platform before proceeding with analyzing human subjects samples, we induced PLK4 overexpression with doxycycline treatment, then captured cells by CD45 exclusion and EpCAM selection using the VERSA platform, and stained for pericentrin and centrin (Fig. 3A). While there remains a significant positive correlation between all pericentrin measurements with the number of centrin foci (Fig. 3B–E), after processing through the VERSA, pericentrin area x intensity and pericentrin area show the strongest correlations with centrin foci (*R* ^2^ = 0.59 and 0.57, respectively).

We then piloted our method with a small number of subjects to ensure that we could capture CTCs and detect pericentrin (Fig. S1). We also established objective numerical cutoffs for expression of pan‐cytokeratins and exclusion channels that would define CTCs versus PBMCs (Fig. S1). Furthermore, we performed the same assay with blood from three healthy individuals and were unable to detect CTCs.

To assess the sensitivity of our assay, we spiked MCF7 breast cancer cells into blood from healthy donors. The VERSA method was able to capture approximately 95% of MCF7 cells, and after device fixation, permeabilization, staining, and washing, we identified approximately 30% of MCF7 cells (Fig. S2).

### 3.4 Centrosome amplification is a frequent event in metastatic breast cancer CTCs

**Fig. 4:**
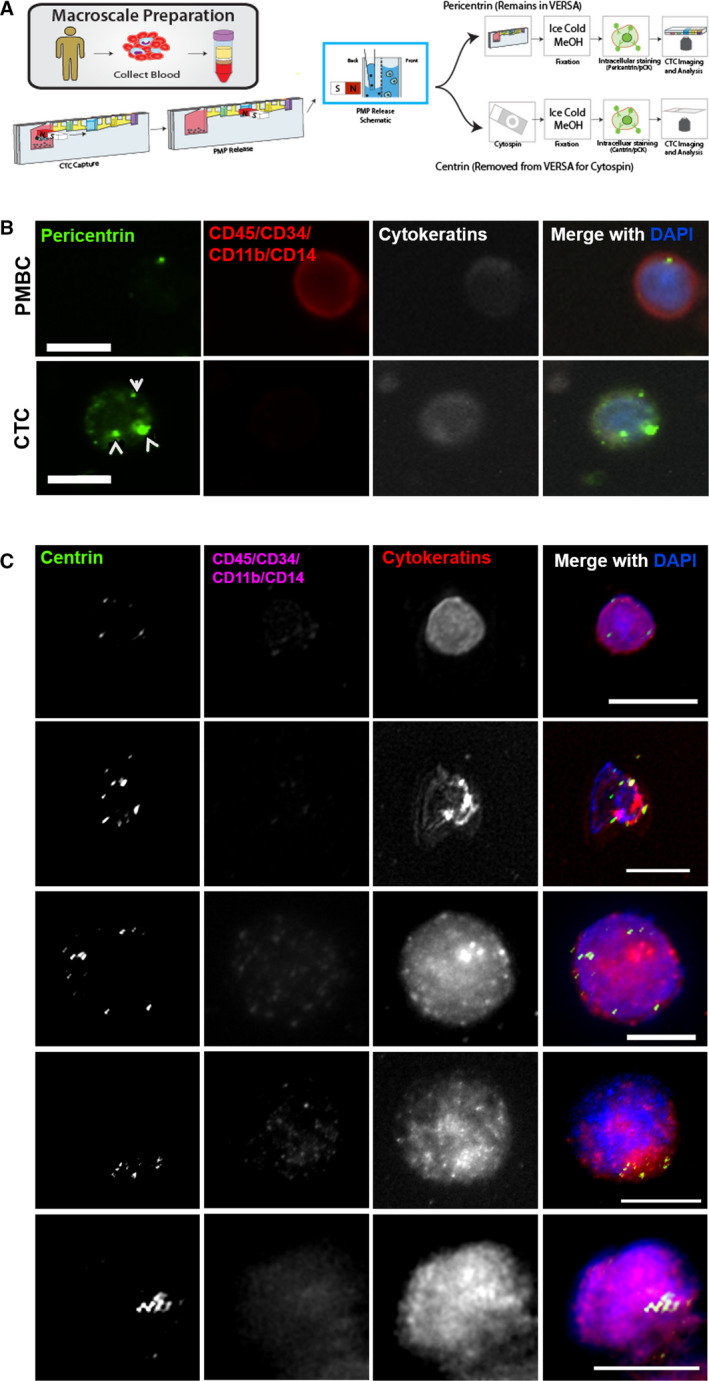
Method for assessing CA in CTCs. (A) Schematic diagram of the VERSA platform. Briefly, the blood samples were processed with Ficoll gradient to isolate all the nucleated cells. Nucleated cells were further depleted of CD45‐positive cells using a magnetic column and transferred to a VERSA microfluidic device for EpCAM capture. Samples were then divided in half for pericentrin staining in VERSA or centrin staining after cells are cytospun onto coverslips. For pericentrin quantification in CTCs, the entire sample was imaged, and CTCs were detected automatically using preset thresholds for the expression of cytokeratin and exclusion channel (CD45/CD34/CD11b/CD14). For quantification of centrin foci, the other half of the sample was cytospun onto coverslips, fixed in methanol, and stained. CTCs were manually identified, and centrioles were manually counted. (B,C) Representative images of pericentrin (B) and centrin (C) staining in CTCs and PBMCs isolated from subjects with metastatic breast cancer. Scale bars = 10 μm.

**Fig. 5:**
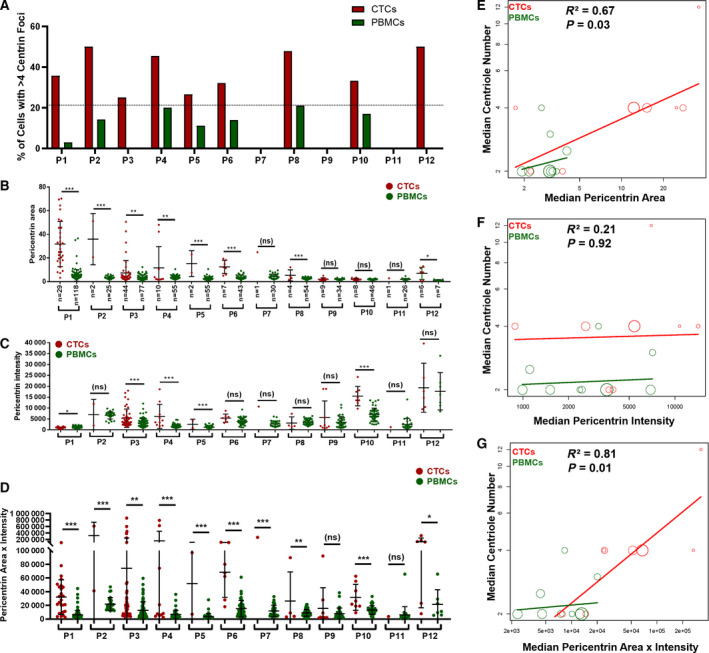
CA is prevalent in CTCs from subjects with metastatic breast cancer. (A) Quantification of the percentage of cells with CA, defined as> 4 centrin foci per cell. A dotted line is drawn at 21%, above which is considered a cancer with CA. (B–D) Quantification of pericentrin area (B), pericentrin intensity (C), and pericentrin area x intensity (D) in CTCs (red dots) versus matched PBMCs (green dots) for each subject. Each dot represents one cell, bars represent mean ± SD, and statistical significance is indicated as follows: *p < 0.05; **p < 0.01; ***p < 0.001; ns, not significant. (E–G) Correlation of centrin foci with pericentrin area (E), pericentrin intensity (F), or pericentrin area x intensity (G). Each dot represents one subject, and the size of the dots is proportional to the number of cells analyzed for that subject.

To measure CA in breast cancer, we collected blood samples from 12 female subjects with metastatic breast cancer. Subjects characteristics, such as age, time since diagnosis, tumor features (e.g., subtype, stage, grade), and lines of therapy are summarized in Table 1. The VERSA platform was used to capture EpCAM‐positive metastatic breast cancer CTCs and assess centrin and pericentrin using the workflow shown in Fig. 4A. Matched PBMCs served as a control. Manually scored centrin foci were used as a direct measure of CA (Fig. 4B‐C, Fig. S3). The number of centrin foci per CTC ranged from 0 to 16, with 9 of 11 samples showing evidence of CA (centrin foci> 4; Fig. S3). The percentage of CTCs with CA varied from 0% to 50% with an average of 28.8% (Fig. 5A). PBMCs are not expected to exhibit CA, and the greatest percentage of PBMCs with> 4 centrin foci was 21% (subject 8). Therefore, samples in which> 21% of cells had> 4 centrin foci were considered to exhibit CA. Based on this threshold, CA was observed in subjects 1, 2, 3, 4, 5, 6, 8, 10, and 12 (Fig. 5A).

**Table 1:** Characteristics of breast cancer subjects

Subject characteristics	N = 12
Age, median (range), years	62 (51–75)
Time since diagnosis (range), years	8 (3–18)
Primary disease	
Histology type, % (*N*)	
IDC	75 (9)
ILC	25 (3)
Tumor stage, % (*N*)	
n/a	8 (1)
I	8 (1)
II	42 (5)
III	17 (2)
IV	25 (3)
Tumor grade, % (*N*)	
n/a	8 (1)
1	8 (1)
2	42 (5)
3	42 (5)
Tumor size, median (range), cm	2 (1.1–11)
Lymph node status positive, % (N)	58 (7)
ER positive, % (N)	83 (10)
PR positive, % (N)	67 (8)
HER‐2 positive, % (N)	25 (3)
Metastatic disease	
Metastatic sites, % (N)	
Bone	67 (8)
Liver	33 (4)
Brain	8 (1)
Lung	42 (5)
Other	42 (5)
Lines of therapy, median (range)	7 (2–9)

Because CA has been reported to promote invasive features (Denu *et al.*, 2016; Godinho *et al.*, 2014), we hypothesized that the degree of CA would correlate with the absolute number of CTCs detected for each subject. However, a strong correlation between CA and number of CTCs was not identified (Fig. S4).

In many subjects, CTCs showed significantly greater pericentrin area (Fig. 5B), pericentrin intensity (Fig. 5C), and pericentrin area x intensity (Fig. 5D) compared to their matched PBMCs, consistent with CA. While both pericentrin area and pericentrin area × intensity positively correlated with CA, pericentrin area × intensity correlated particularly well with number of centrin foci (R^2^ = 0.81, Fig. 5E–G). In fact, pericentrin area × intensity was able to predict an increase in centrin foci accurately in 100% (11/11) of subjects for which more than one CTC could be scored; this excludes subject 7, for which only a single CTC was evaluable. We conclude that automated imaging of pericentrin centrin area x intensity is a valid surrogate measure of CA in metastatic breast cancer.

## 4 Discussion

CA has been observed in wide variety of human cancers (Denu *et al.*, 2016; Giehl *et al.*, 2005; Kramer *et al.*, 2005; Lingle *et al.*, 1998; Pihan *et al.*, 1998) and can promote CIN and aneuploidy (Ganem *et al.*, 2009; Silkworth *et al.*, 2009) and metastasis (Godinho *et al.*, 2014). In the present report, we directly measure CA in CTCs from subjects with metastatic breast cancer and find that it is strikingly common, occurring in 75% of subjects with metastatic breast cancer and up to 50% of CTCs per subject. To the best of our knowledge, this is the first report demonstrating CA in cancer CTCs.

Though number of centrin foci is the gold standard measure of CA, measurements of centrin are technically challenging under optimal experimental conditions and were incompatible with automated analysis of CTCs, making centrin foci impractical as a clinical assay. However, pericentrin staining is robust. We therefore validated pericentrin as a surrogate marker of the number of centrin foci and optimized pericentrin staining in our VERSA‐based, automated analysis. We find that pericentrin area x intensity accurately predicts centriole amplification in 100% of subjects for which greater than one CTC was evaluable. As this high‐throughput assay can readily analyze patient samples, this permits widespread testing of CA.

As CA is known to promote invasive features (Denu *et al.*, 2016; Godinho *et al.*, 2014), we hypothesized that the degree of CA would correlate with the absolute number of CTCs detected for each subject. Furthermore, in a study where SUIT‐2 human pancreatic cancer cells were xenografted into nude mice, CA was more prevalent in metastatic foci than the original xenograft (Shono *et al.*, 2001). However, we did not observe a strong correlation between CA and the number of CTCs. One potential explanation is the small number of subjects in our study, which may have limited the power to detect such a correlation. A second explanation is that many other factors may contribute more to invasiveness than CA alone. Alternatively, CA may be more prevalent in the primary tumor from which the CTCs are derived, so it may not be expected that the CTCs themselves would have more CA. Evidence for this explanation comes from a previous study showing that CA promotes tumor cell invasiveness in a nonautonomous fashion (Ganier *et al.*, 2018). A final possibility is that CA is not as strong at promoting metastatic dissemination as predicted by preclinical models.

One limitation of this study is the small number of recruited subjects, requiring validation in a larger sample size. However, this study provides proof of principle that CA is detectable in CTCs, and pericentrin area × intensity can be used as an automated, quantitative biomarker in future studies.

In the future, our reported assay may be useful as a predictive biomarker. For example, since CIN may predict response to taxane chemotherapy (Funk *et al.*, 2016; Lee *et al.*, 2013; Rajagopalan and Lengauer, 2004; Tanaka and Hirota, 2016; Weaver, 2014), measuring CA in CTCs may be useful in predicting taxane sensitivity in patients with metastatic breast cancer.

## 5 Conclusion

In conclusion, we report the presence of frequent CA events in EpCAM‐captured CTCs from subjects with metastatic breast cancer. This assay will be useful for longitudinal monitoring of CA in cancer patients and in prospective clinical trials for assessing therapeutic response to agents whose efficacy is affected by CA or CIN.

## Conflict of interest

JM Lang has ownership interest (including patents) in Salus Discovery. Other authors disclosed no potential conflicts of interest.

## Author contributions

JML, MEB, and BAW conceived the project and secured funding. AS and RAD conceived the project and acquired the data. SKW, JMS, JS, and TW acquired the data. In addition, all authors interpreted the data and contributed to the manuscript drafting, revision, and approval.

## Supporting information


**Figure S1.** Capture of EpCAM‐positive CTCs from subjects with metastatic breast cancer.
**Figure S2.** MCF7 spike‐in to quantify the assay sensitivity.
**Figure S3.** Quantification of centrin foci in CTCs.
**Figure S4.** CA does not correlate well with absolute number of CTCs captured.
