# Supplementary material for: Centrosome amplification is a frequent event in circulating tumor cells from subjects with metastatic breast cancer
Source: Mol Oncol. 2020 May 19;14(8):1898–909. doi: 10.1002/1878-0261.12687 (PMC7400789; doi:10.1002/1878-0261.12687)

**SUPPORTING INFORMATION**

**Centrosome amplification is a frequent event in circulating tumor cells from subjects with metastatic breast cancer**

Ashok Singh^1^*****, Ryan A. Denu^2^*****, Serena K. Wolfe^1^, Jamie M. Sperger^1^, Jennifer Schehr ^1^, Tessa Witkowsky^1^, Karla Esbona^1^, Richard J. Chappell^3^, Beth A. Weaver^1,4^, Mark E. Burkard^1,2^, Joshua, M. Lang^1,2^

**Affiliation**: ^1^Carbone Cancer Center, ^2^Department of Medicine, Division of Hematology/Oncology, ^3^Departments of Statistics and of Biostatistics & Medical Informatics, ^4^Department of Cell and Regenerative Biology and Department of Oncology/McArdle Laboratory for Cancer Research, University of Wisconsin-Madison, Madison, WI, 53705

***Contributed equally**

**Corresponding Author:**

Joshua, M. Lang, Department of Medicine, Carbone Cancer Center, University of Wisconsin- Madison 1111 Highland Avenue, WIMR 7151, Madison, WI 53705, Phone: 608-262-0705 Fax: 608-265-0614 Email: [jmlang@medicine.wisc.edu](mailto:jmlang@medicine.wisc.edu)

**Supplemental Figure 1: Capture of EpCAM-positive CTCs from subjects with metastatic breast cancer.**

Representative images of PBMCs versus CTCs from subjects with metastatic breast cancer to ensure that CTCs could be captured and pericentrin detected. Scale bars = 10μm.


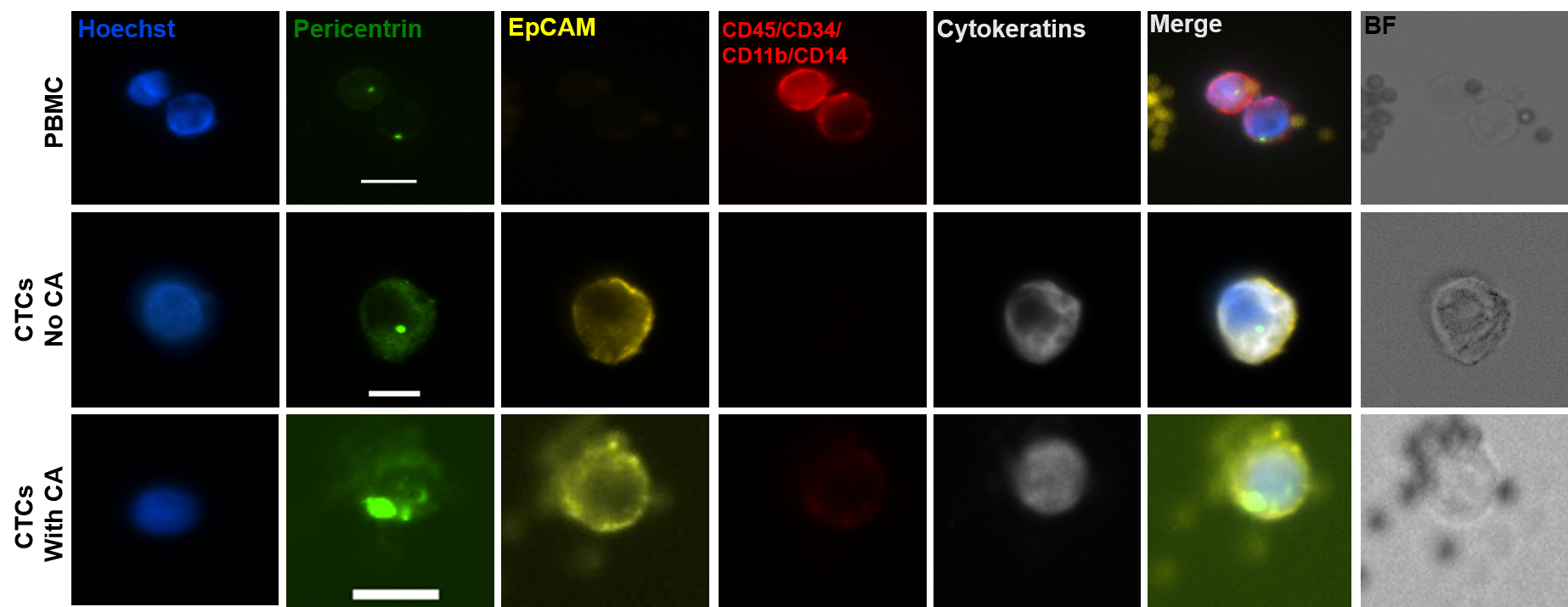


**Supplemental Figure 2: MCF7 spike-in to quantify the assay sensitivity.**

**(A)** Average MCF7 capture efficiency with EpCAM labeled PMPs. **(B)** Percent of MCF7 cells identified after cell capture, fixation, permeabilization, staining and washing of MCF7 cells spiked into PBMCs from healthy donor blood. Approximately 5, 100, and 500 MCF7 cells were spiked. **(C)** Representative images of PBMCs and MCF7 cells from spike-in experiments.

**
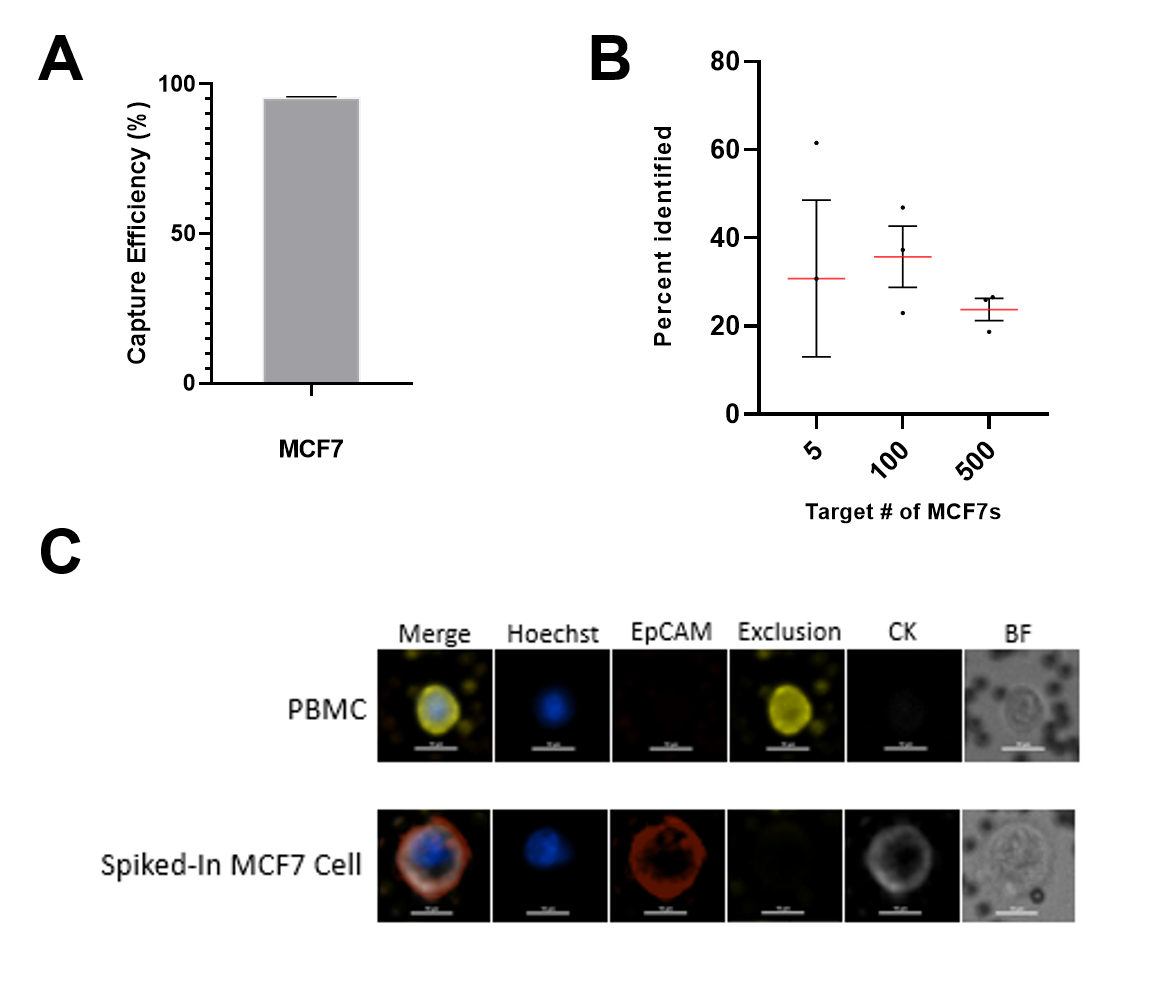
**

**Supplemental Figure 3: Quantification of centrin foci in CTCs.**

Quantification of centrin foci per cell (CTCs and PBMCs) in each subject in the study. A dotted line is drawn at a centriole count of 4, above which is considered “centrosome amplification.”

**
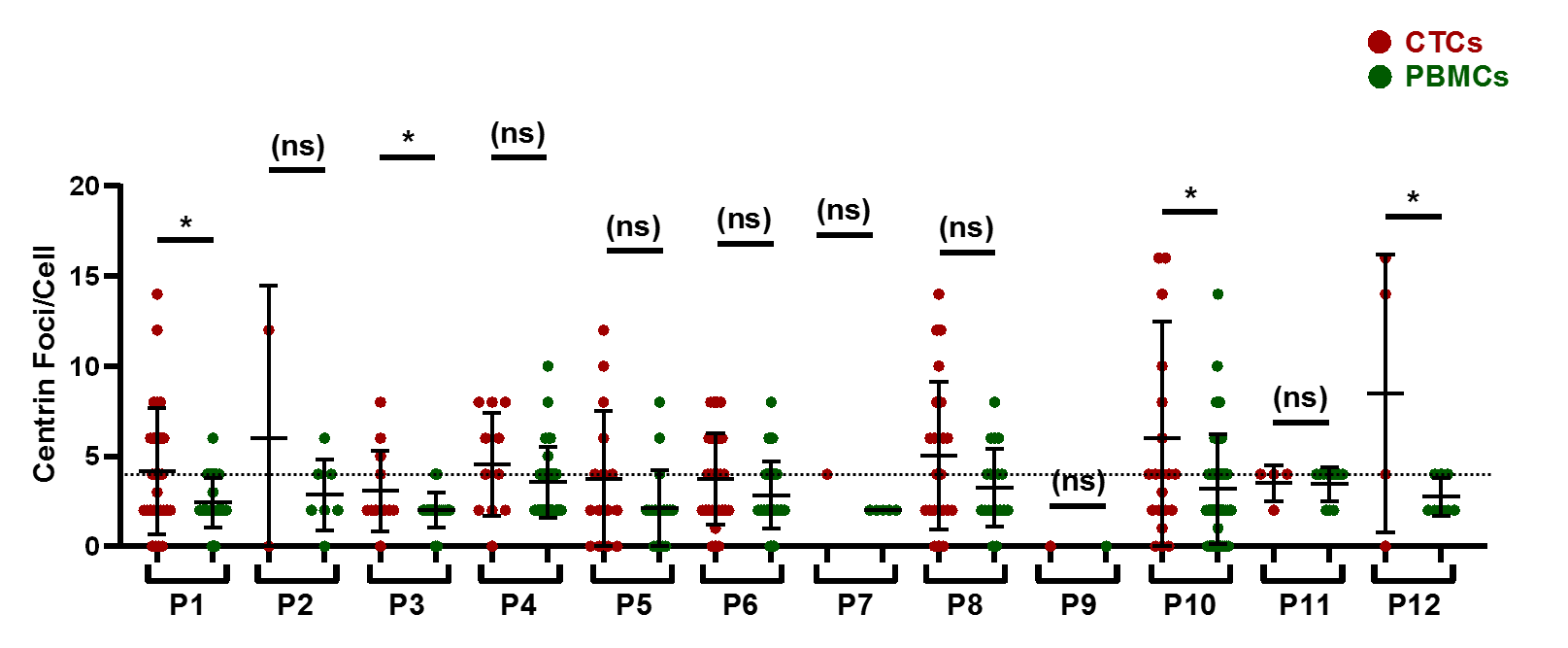
**

**Supplemental Figure 4: CA does not correlate well with absolute number of CTCs captured.**

Correlation of centrin foci **(A)**, pericentrin area **(B)**, and pericentrin area **(C)** with CTC number.


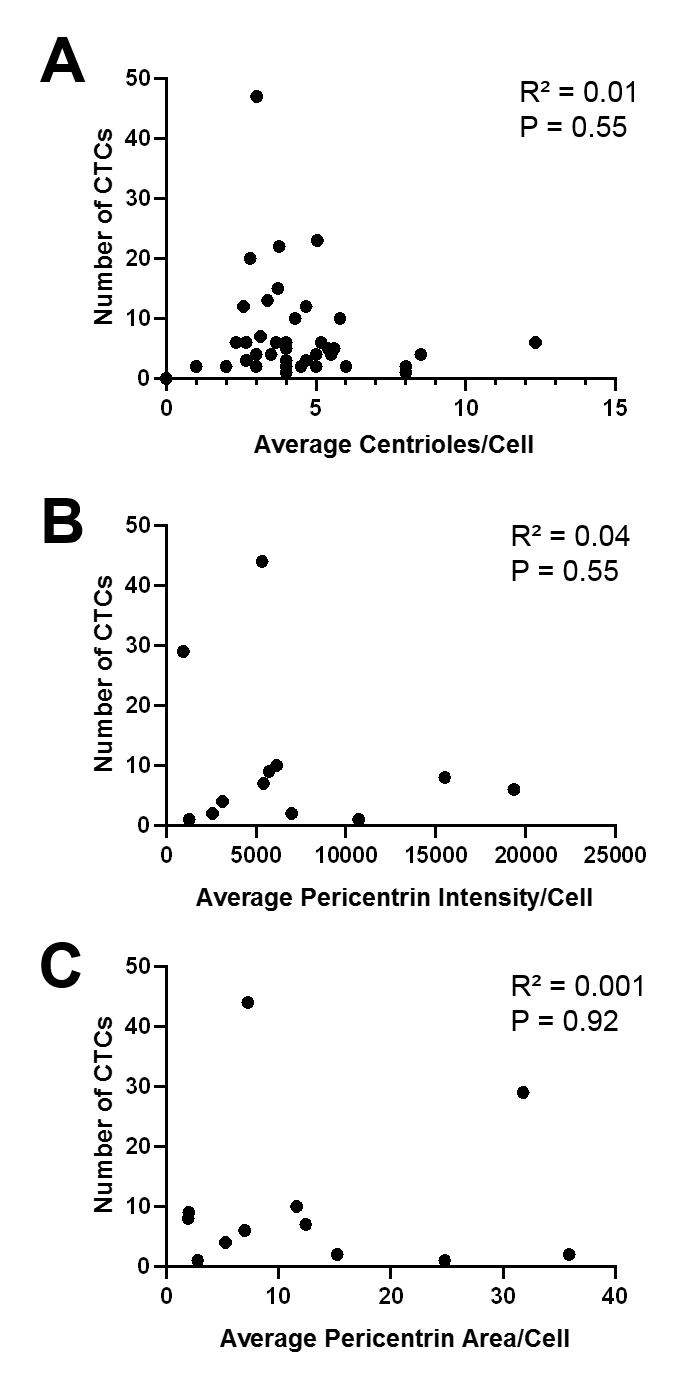

Supplement: Supplementary file 1 — Figure S1. Capture of EpCAM‐positive CTCs from subjects with metastatic breast cancer. Figure S2. MCF7 spike‐in to quantify the assay sensitivity. Figure S3. Quantification of centrin foci in CTCs. Figure S4. CA does not correlate well with absolute number of CTCs captured. [file MOL2-14-1898-s001.docx]
